# Skeletal and Dentoalveolar Effects of the SOCIA III Appliance in Patients with Pseudo-Class III Malocclusion

**DOI:** 10.3390/dj13090427

**Published:** 2025-09-15

**Authors:** Mauro Lorusso, Giovanna Jie Wang, Michele Tepedino, Angela Pia Cazzolla, Fariba Esperouz, Lucio Lo Russo, Domenico Ciavarella

**Affiliations:** 1 Department of Clinical and Experimental Medicine, Dental School of Foggia, University of Foggia, 71122 Foggia, Italy; giovanna.wang@unifg.it (G.J.W.); angelapia.cazzolla@unifg.it (A.P.C.); fariba.esperouz@unifg.it (F.E.); lucio.lorusso@unifg.it (L.L.R.); domenico.ciavarella@unifg.it (D.C.); 2 Department of Biotechnological and Applied Clinical Sciences, Dental School of L’Aquila, University of L’Aquila, 67100 L’Aquila, Italy; michele.tepedino@univaq.it

**Keywords:** SOCIA III appliance, pseudo-class III malocclusion, atypical swallowing, growing patients

## Abstract

**Objective:** This study aimed to assess the dentoskeletal and postural modifications induced by treatment with the Swallowing Occlusal Contact Intercept Appliance (SOCIA III) in patients with pseudo-Class III malocclusion. The main hypothesis was that treatment with the SOCIA appliance induces significant skeletal and dentoalveolar changes in growing patients with pseudo-Class III malocclusion. **Methods:** Fifty-two pseudo-Class III malocclusion patients with a mean age of 8.5 were analyzed and compared with fifty-two untreated patients. Cephalometric evaluations were carried out at baseline (T0) and at the end of treatment. The cephalometric analysis comprised sagittal measurements (SNA, ANB, WITS, CB, ACB), dental variables (UI-PP, LI-MP, OVERJET, OVERBITE), and postural parameters (SNBa and cranio-cervical angle of the maxilla). Within-group comparisons at baseline (T0) and post-treatment (T1) were performed using the paired *t*-test or the Wilcoxon signed-rank test. Inter-group comparisons were based on the differences in variables between the two time points (ΔT1–T0), using the independent samples *t*-test or the Mann–Whitney U test. **Results:** At the end of treatment with the SOCIA appliance, significant improvements were observed in the ANB angle (0.61° to 2.06°, *p* = 0.001), SNA (81.56° to 83.26°, *p* = 0.006), WITS appraisal (−0.75 mm to 1.24 mm, *p* = 0.001), ACB angle (9.35 mm to 11.45 mm, *p* = 0.05), CB (64.78 mm to 65.51 mm, *p* = 0.03), and SNBa angle (127.57° to 129.14°, *p* = 0.01), as well as in overjet (0.62 mm to 3.60 mm, *p* = 0.001) and overbite (0.29 mm to 1.82 mm, *p* = 0.001). **Conclusions:** The SOCIA III appliance is effective in correcting pseudo-Class III malocclusion in growing patients by improving skeletal and dentoalveolar relationships.

## 1. Introduction

The prevalence of Class III malocclusion ranges from 0% to 26% worldwide [1]. In European populations, it is estimated at approximately 5%, while in East Asian populations it varies between 13% and 19% [2].

The etiology of Class III malocclusion is complex, involving a dynamic interplay between genetic predisposition and environmental influences. Genetics plays a decisive role in determining skeletal growth patterns, yet several environmental factors may substantially affect the expression and severity of the condition. Prolonged deleterious oral habits, such as thumb-sucking or excessive pacifier use, can interfere with normal maxillomandibular development, thereby contributing to abnormal skeletal relationships [3]. Chronic mouth breathing secondary to nasal obstruction may alter tongue posture and restrict maxillary growth, while atypical swallowing, characterized by abnormal tongue dynamics during deglutition, can negatively affect both dental alignment and skeletal morphology [4].

In addition, postural deviations and muscular imbalances may further disturb mandibular positioning, exacerbating interarch discrepancies [5]. These environmental factors often act synergistically with genetic susceptibility, leading to a wide spectrum of clinical presentations.

Several studies have reported genetic alterations associated with hereditary forms of Class III malocclusion [3,6]. Nevertheless, the precise role of these genes and their mutations in determining susceptibility and clinical expression remains unclear [7], suggesting that genetic factors alone cannot fully account for the heterogeneity of the condition.

Skeletal Class III malocclusion generally arises from maxillary retrusion, mandibular protrusion, or a combination of these skeletal discrepancies [6,8]. Patients with Class III malocclusion often present a combination of dental and skeletal discrepancies, including dentoalveolar imbalances, sagittal disharmony, and vertical growth alterations [9].

Furthermore, inadequate maxillary growth in this population has been associated with an increased risk of canine impaction, further complicating orthodontic management [10,11].

Early diagnosis, preferably during the late primary or early mixed dentition stage, is essential, since orthopedic and orthodontic interventions initiated at these phases are significantly more likely to achieve favorable and stable outcomes [12].

Accurate diagnosis requires a thorough evaluation of the clinical and cephalometric features of the malocclusion. According to Tweed’s classification [13], Class III malocclusion can be divided into two main categories:

- Category A: Pseudo-Class III malocclusion, characterized by a normally shaped mandible with a functional forward displacement.
- Category B: Skeletal Class III malocclusion, associated with a prognathic mandible and/or a retrognathic maxilla.

This distinction is clinically relevant, as the therapeutic approach differs substantially between the two forms. Clinically, pseudo-Class III malocclusion is characterized by anterior crossbite, a Class I molar relationship or a mild mesial shift (up to ¼ cusp, approximately 1–2 mm), and a Wits appraisal typically ranging between +2.0 mm and −2.0 mm. Maxillary and mandibular lengths are usually within normal limits, while the lower facial height is often normal or slightly reduced [14]. Patients frequently exhibit retroclined maxillary incisors, proclined mandibular incisors, and occasionally diastemas [15,16]. Timely intervention is essential to prevent the progression of malocclusion into more severe and complex forms that are challenging to manage [17].

Orthodontic intervention in these cases primarily targets the dentoalveolar component, aiming to correct the anterior crossbite and reestablish a correct Class I molar occlusion.

In pseudo-Class III malocclusion, orthodontic intervention generally targets the dentoalveolar component, with the primary goal of correcting anterior crossbite and reestablishing a stable Class I molar relationship.

Management becomes more complex when pseudo-Class III malocclusion is associated with atypical swallowing. In these cases, the tongue acts as an activator, exerting anterior pressure on the mandible and aggravating the malocclusion [18]. Consequently, it is crucial to correct atypical swallowing through the use of a device that promotes proper tongue repositioning. To date, no studies have investigated the effects of a functional appliance specifically designed for the treatment of pseudo-Class III malocclusion associated with atypical swallowing.

The present study aimed to evaluate the dentoskeletal and postural effects of the Swallowing Occlusal Contact Intercept Appliance (SOCIA III) in growing patients with pseudo-Class III malocclusion and atypical swallowing. The main hypothesis states that treatment with the SOCIA appliance induces significant skeletal and dentoalveolar modifications in growing patients with pseudo-Class III malocclusion.

## 2. Materials and Methods

This retrospective study was conducted according to the Strengthening the Reporting of Observational Studies in Epidemiology (STROBE) guidelines [19]. All methods specified in the study design complied with the Declaration of Helsinki and received approval from the Ethics Committee of the University. All participants’ parents provided written informed consent.

Patients were eligible for the study if they met the following criteria:

- A wits appraisal < 0 mm;
- An overjet < 1 mm;
- The divergence angle within the normal range;
- Presence of atypical swallowing pattern;
- The ANB angle within the normal range;
- Mixed dentition;
- Skeletal age corresponding to stages CS2 to CS3, as determined by the cervical vertebral maturation (CVM) method.

Patients were excluded from the study if they presented the following conditions:

- Class II malocclusion;
- Skeletal class III malocclusion;
- Bilateral crossbite;
- Systemic or oral diseases;
- Missing teeth;
- Congenital malformations;
- A history of prior orthodontic treatment.

A power analysis [20] was performed using G*Power 3.1.9.2 software (Franz Faul, Universität Kiel, Germany). Based on a predicted medium effect size (0.5), with a *t*-test, a power of 0.90 and alpha = 0.05, a minimum of 44 subjects would be needed for each group.

The study population included two groups: Group S, consisting of patients treated with the SOCIA appliance, and Group C comprised an untreated control group.

A total of 52 patients with pseudo-Class III malocclusion (28 males and 24 females; mean age: 8.5 ± 0.4 years) were included in Group S. These patients were treated with the SOCIA appliance for a duration of 16 months (range: 14–18 months). Treatment was concluded upon achieving a Class I molar and canine relationship. This group was retrospectively selected from individuals treated at the University Department of Orthodontics in chronological order between January 2022 and March 2024.

The diagnosis of atypical swallowing was established by instructing patients to perform five consecutive swallows at 5 s intervals, in order to evaluate tongue positioning during the act of deglutition.

Group C included 52 untreated patients (26 males and 26 females; mean age: 8.7 ± 0.3 years). These individuals were selected from the Michigan Medical Library database and matched to Group S by age, sex and cephalometric characteristics for comparative purposes.

### 2.1. SOCIA Appliance

The SOCIA III is a functional appliance custom-made for each patient. It has no dental retention; instead, the activity of the masticatory muscles holds the appliance in the correct position. It is used to rehabilitate atypical swallowing, specifically addressing a low tongue position during the act of swallowing. Atypical swallowing is defined as the persistence of an infantile swallowing pattern without the normal transition to the adult type. This condition is characterized by an altered tongue posture, where the tip of the tongue rests against the palatal surfaces of the anterior teeth or protrudes between the dental arches instead of positioning against the palate. Additionally, the tongue dorsum tends to curve downward, with its base making contact with the posterior palate and the anterior pharyngeal wall.

The appliance consists of an acrylic palatal body featuring a lingual plane tilted at 60° relative to the occlusal plane, terminating in a hole near the palatal spot.

The vestibular shields are positioned 4 mm buccal to the deciduous molars, with metallic posterior bite-blocks embedded within them for added stability. The upper labial arch is incorporated into the acrylic labial shields, forms a loop at the level of the upper canine, and extends downward to the middle third of the labial surface of the lower anterior teeth. Internally, a steel arch originates from the acrylic lingual shield and projects into the lingual space of the upper incisors.

The vestibular components are connected by a 1.0 mm labial wire that follows the contour of the dental arch and a 1.1 mm wire that crosses the occlusal plane, anchoring into the palatal acrylic body.

Patients were instructed to wear the SOCIA appliance for 16 h per day, during the night and afternoon, removing it only for eating and brushing their teeth.

**Figure 1:**
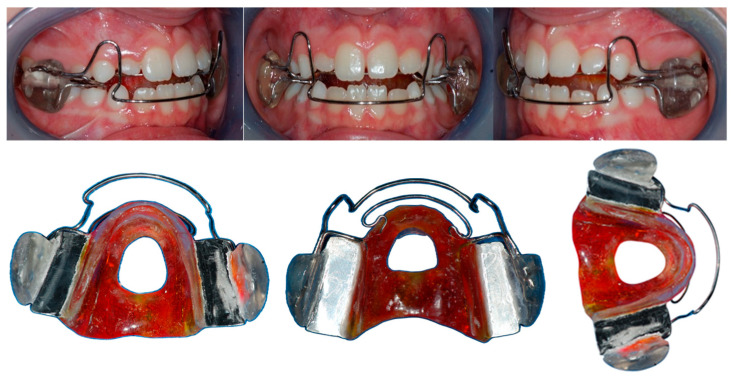
The SOCIA appliance: intraoral, frontal and lateral view.

Compliance was clinically evaluated through follow-up visits conducted every two weeks, complemented by parental supervision. All patients met the inclusion criteria by demonstrating satisfactory compliance throughout the treatment period. Figure 1 presents the device as worn by a patient, as well as a frontal view of the appliance.

### 2.2. Cephalometric Analysis

Lateral cephalometric radiographs were obtained at two time points: T0 (beginning of treatment) and T1 (end of treatment). All measurements were performed on digitized cephalograms by a single operator with extensive training in digital cephalometric analysis. The same operator conducted the cephalometric assessments to ensure consistency. To minimize bias, the measurements were carried out in a blinded manner. The orthodontist responsible for the analysis was not involved in the clinical management of the patients, and all data were anonymized prior to evaluation.

The cephalometric analysis included sagittal (SNA, ANB, WITS, CB, ACB), dental (UI-PP, LI-MP, overjet, overbite), and postural parameters (CC Maxilla, SNBa).

**Figure 2:**
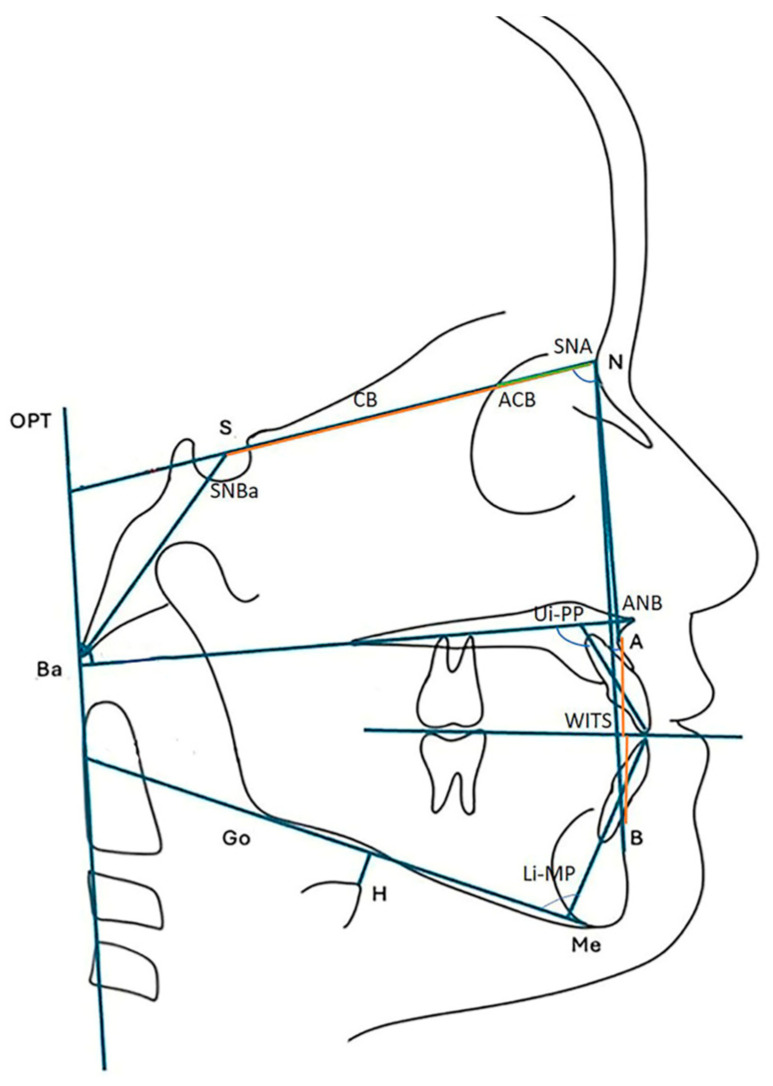
Anatomical landmarks and reference lines used in cephalometric analysis.

The anatomical landmarks and reference lines used in the cephalometric analysis are illustrated in Figure 2 and detailed in Table 1.

### 2.3. Statistical Analysis

Statistical analysis was conducted using GraphPad Prism software (version 6.04 for Windows, GraphPad Software, La Jolla, CA, USA). Descriptive statistics (Table 2) were computed to summarize the data and provide a clearer interpretation of the results. The Shapiro–Wilk test was applied to all variables to assess whether the data followed a normal distribution.

For variables demonstrating a normal distribution, intra-group comparisons between the baseline (T0) and post-treatment (T1) values were performed using the paired *t*-test. In cases where normality was not confirmed, the Wilcoxon signed-rank test was used instead (Table 3).

Inter-group comparisons were based on the differences in change between the two time points (ΔT1–T0). These comparisons were performed using either the independent samples *t*-test for normally distributed data or the Mann–Whitney U test for non-normally distributed data (Table 4).

The level of statistical significance was set at *p* < 0.05.

To minimize random errors, all skeletal and dental cephalometric measurements were repeated after a 15-day interval by a single calibrated examiner. The random error of each measurement was calculated using Dahlberg’s formula (*S* = √∑ *d*^2^/2*N*), where *d* is the difference between the first and second measurements and *N* the number of radiographs evaluated [21,22]. The random error ranged between 0.08 and 0.1 mm for linear measurements and between 0.2 and 0.3 degrees for angular measurements.

## 3. Results

Table 2 presents the descriptive statistics of the sample.

### 3.1. Intra-Group Comparison

In the SOCIA group, significant improvements were observed in several sagittal parameters (Table 3). The ANB angle increased from 0.61° (95%CI 0.53, 0.69) to 2.06° (95%CI 1.98, 2.14), *p* = 0.001, the SNA angle increased from 81.56° (95% CI 81.2, 81.92) to 83.26° (95% CI 82.97–83.55), *p* = 0.006 and the WITS appraisal improved from −0.75 mm (95% CI −0.99, −0.51) to 1.24 mm (95%CI 1.14, 1.34), *p* = 0.001. A significant increase in cranial base length was also observed, both in CB from 64.78 mm (95%CI 63.72, 65.84) to 65.51 mm (95%CI 64.70, 66.32), *p* = 0.03 and ACB from 9.35 mm (95%CI 8.58, 10.12) to 11.45 mm (95%CI 10.69, 12.21), *p* = 0.05.

Postural changes were highlighted by a significant increase in the SNBa angle from 127.57° (95%CI 126.01, 129.13) to 129.14° (95%CI 127.47, 130.81), *p* = 0.01.

Dental parameters showed significant correction of the anterior crossbite. Overjet increased from 0.62 mm (95%CI −0.06, 1.3) to 3.6 mm (95%CI 3.31, 3.89), *p* = 0.001, and overbite improved from 0.29 mm (95%CI −0.09, 0.67) to 1.82 mm (95%CI 1.6, 2.04) *p* = 0.001.

Clinical Implications:

The treatment resulted in significant improvements in skeletal and dental relationships, primarily through maxillary advancement, as evidenced by increases in ANB, SNA, and WITS values. Increases in cranial base length and SNBa angle suggest associated skeletal and postural adaptations. Vertical facial growth remained stable, and dental parameters showed effective correction of anterior crossbite, with marked improvements in overjet and overbite.

### 3.2. Inter-Group Comparison

Statistical analysis compared the T1-T0 differences between the groups (Table 4). The SOCIA group showed a greater increase in ANB (+1.46° vs. −1.20°, *p* = 0.001), WITS (+2.0 mm vs. −0.9 mm, *p* = 0.001), and ACB (+2.10 mm vs. +1.26 mm, *p* = 0.01). A significant difference was also observed for SNBa (+1.57° in SOCIA vs. −1.14° in controls, *p* = 0.002), and for lower incisor inclination (LI-MP decreased by 1.95° in SOCIA vs. increased by 1.39° in controls, *p* = 0.01).

Clinical Implications:

SOCIA treatment resulted in significantly greater improvements in skeletal and dental parameters compared to controls, including enhanced maxillo-mandibular relationships, increased cranial base growth, and favorable lower incisor inclination, underscoring its effectiveness in orthodontic correction.

## 4. Discussion

This study evaluated the dentoskeletal and postural effects of the SOCIA III appliance in patients with pseudo-Class III malocclusion. Functional appliances remain a cornerstone in the early management of skeletal discrepancies during craniofacial development, with treatment outcomes being most favorable when initiated in the early mixed dentition. The SOCIA III appliance (Swallowing Occlusal Contact Intercept Appliance) was developed to correct anterior crossbite by promoting neuromuscular balance and guiding the development of the maxilla and mandible through muscular and functional stimuli [23].

Several orthopedic appliances have been proposed for the early treatment of Class III malocclusion. The Reverse Twin-Block, for example, is indicated for patients with anterior crossbite, negative overjet, or an edge-to-edge incisal relationship. Its reverse occlusal angulation directs occlusal forces toward maxillary advancement while simultaneously restraining mandibular forward growth [24]. However, its effects are mainly dentoalveolar, typically involving proclination of the maxillary incisors and retroclination of the mandibular incisors, while skeletal changes are minimal and limited to mild mandibular rotation and a slight increase in vertical facial dimension [25].

Similarly, the Frankel Type III appliance, introduced in the early 1970s, relies on labial pads and buccal shields to reduce perioral muscular pressure, thereby stimulating transverse and sagittal maxillary growth as well as basal bone development [26].

Its skeletal effects include downward and backward mandibular rotation and modest forward stimulation of maxillary growth, while dentoalveolar adaptations consist mainly of maxillary incisor proclination and mandibular incisor retroclination [27].

Ulgen and Firatli reported a significant increase in ANB values in patients treated with Frankel III, largely due to decreased SNB associated with backward mandibular rotation [28]. The effects of these two functional appliances are predominantly dentoalveolar, characterized by changes in the inclination of the upper and lower incisors, accompanied by downward and backward mandibular rotation. In contrast to these appliances, SOCIA therapy induces minimal dentoalveolar modifications, as tongue repositioning eliminates aberrant anterior pressure on the incisors. The main therapeutic benefit derives from stimulation of maxillary growth, achieved both through the physiological action of the tongue acting as an activator and through the stabilizing effect of posterior bite blocks, which together restore balanced neuromuscular function.

### 4.1. Intra-Group Changes

SOCIA therapy produced significant improvements in multiple sagittal skeletal parameters. The SNA angle increased significantly (from 81.56° to 83.26°, *p* = 0.006), indicating anterior displacement of the maxilla. While SNA may be influenced by both positional and dentoalveolar factors, the stability of maxillary incisor inclination after treatment rules out dental compensation as a major contributor. Furthermore, given the absence of an anterior orthopedic force vector, such as that delivered by facemask therapy, the observed increase is more likely attributable to functional stimulation. Specifically, the tongue, guided by the appliance into a corrected posture, exerted sustained physiological pressure on the premaxillary region, thereby stimulating bone apposition and anterior skeletal growth. This interpretation is consistent with the hypothesis that functional rehabilitation, particularly in patients with atypical swallowing, can enhance maxillary development during growth. Comparable results have been reported with Frankel III and Class III elastodontic appliances, which act by reducing neuromuscular constraints and promoting forward maxillary displacement [29,30].

The ANB angle increased from 0.61° to 2.06°, and the WITS appraisal improved from −0.75 mm to +1.24 mm (*p* = 0.001), indicating substantial sagittal correction.

Cranial base measurements CB and ACB also increased (CB: *p* = 0.03; ACB: *p* = 0.05).

Additional evidence of sagittal correction was observed in the ANB angle (from 0.61° to 2.06°) and the WITS appraisal (from −0.75 mm to +1.24 mm; both *p* = 0.001), confirming substantial improvements in maxillo-mandibular relationships [31].

The SNBa angle increased significantly as well (*p* = 0.01), indicating a favorable cranio-cervical adaptation consistent with literature linking mandibular growth direction to cranio-cervical angulation [32,33,34].

Other cephalometric variables remained unchanged, reflecting the specific biomechanical action of the appliance. The maxillary incisor inclination showed no significant variation, in line with SOCIA’s design, which does not include auxiliaries acting directly on the anterior dentoalveolar segment. A lingual inclination of mandibular incisors was observed, likely due to the combined effects of the lingual arch, acting as a stabilizer, and the reduction in tongue thrust facilitated by the resin shield [35].

Dental changes were also clinically relevant. Overjet improved from 0.62 mm to 3.60 mm (p = 0.001), and overbite from 0.29 mm to 1.82 mm (*p* = 0.001), effectively resolving the anterior crossbite and normalizing incisor relationships. These corrections reflect both the elimination of functional mandibular displacement and the re-establishment of appropriate incisal guidance [36,37].

In the control group, the SNA angle exhibited a slight, non-significant increase, while ANB and WITS values decreased. The upper incisors showed modest changes in inclination, and CB and ACB remained essentially unchanged. Overjet and overbite increased only minimally. These findings indicate that physiological growth has a limited impact on the cranial base, and that the pattern of growth may not always be favorable, particularly in patients with dentoskeletal discrepancies that could develop into clinically significant malocclusions requiring orthodontic intervention.

### 4.2. Inter-Group Changes

Direct comparison between the SOCIA-treated group and untreated controls revealed statistically significant differences, confirming the therapeutic impact of the appliance. The SOCIA group demonstrated greater improvements in sagittal parameters, with increases in ANB (ΔANB: +1.46° vs. −1.20°, *p* = 0.001) and WITS appraisal (ΔWITS: +2.00 mm vs. −0.90 mm, *p* = 0.001), underscoring its effectiveness in improving maxillo-mandibular relationships.

An increase in anterior cranial base length (ACB: +2.10 mm vs. +1.26 mm, *p* = 0.01) was also observed in the SOCIA group. This finding suggests that functional rehabilitation through tongue repositioning and neuromuscular rebalancing may extend beyond dental and skeletal corrections to influence cranial base development or remodeling. Consistent with these results, Al Maaitah et al. [31] reported that cranial base length and angulation are modifiable during growth and can influence sagittal jaw relationships. Postural adaptations were further confirmed by significant differences in the SNBa angle (ΔSNBa: +1.57° in SOCIA vs. −1.14° in controls, *p* = 0.002), indicating improved cranio-cervical orientation. Moreover, mandibular incisor inclination (LI-MP) decreased in the SOCIA group (−1.95°) but increased in the controls (+1.39°, *p* = 0.01). This pattern reflects a more favorable dentoalveolar adaptation associated with SOCIA therapy, which likely results from corrected tongue posture rather than from compensatory incisor tipping. The use of the SOCIA appliance induces both skeletal and dental changes, including stimulation of maxillary growth and a reduction in lower incisor inclination, along with an increase in overjet and overbite. These modifications contribute to an improved facial profile, characterized by enhanced maxillary projection and more pronounced labial support [38]. Additionally, treatment appears to promote rebalancing of the perioral musculature, suggesting positive effects not only on dental alignment but also on overall facial harmony [39]. These findings support the efficacy of the SOCIA appliance in addressing both functional and skeletal components of pseudo-Class III malocclusion. By stimulating anterior maxillary growth, correcting anterior crossbite, regulating mandibular positioning, and guiding tongue function, the appliance facilitates neuromuscular rebalancing and promotes harmonious, coordinated craniofacial development.

Several factors, such as socioeconomic variability and differences in facial growth patterns, may have influenced the outcomes of this study and were not fully homogeneous between groups. Importantly, compliance in the SOCIA group was satisfactory, with patients adhering consistently to the prescribed protocol. This observation reinforces the pivotal role of patient cooperation in functional therapies, particularly when removable appliances are involved, as adherence directly affects the likelihood of achieving treatment goals.

The question of long-term stability remains crucial in orthodontic care. Evidence suggests that functional appliances designed to stimulate maxillary growth generally demonstrate favorable stability and a lower risk of relapse [40]. Nonetheless, relapse may still occur as a consequence of persisting deleterious habits, insufficient patient compliance, or genetic predisposition. Notably, relapse is reported more frequently in patients treated with fixed appliances [41], or in cases where the use of archwires leads to modifications of the dental arch form [42]. For these reasons, structured post-treatment follow-up and reinforcement of proper myofunctional behavior are essential. Although long-term evaluation was beyond the scope of this study, future prospective investigations should include extended follow-up periods to assess the durability of SOCIA treatment effects.

### 4.3. Limitations of the Study

This study has inherent limitations that should be acknowledged. First, its retrospective design and reliance on two-dimensional cephalometric analysis. Second, the assessment of atypical swallowing was performed clinically, without electromyographic confirmation, which may reduce the objectivity of functional evaluation. Third, although control subjects were matched for age and sex, they were drawn from a different population, potentially introducing variability due to genetic and environmental differences. These limitations underscore the need for cautious interpretation of the results. Future prospective studies incorporating three-dimensional imaging, standardized functional assessments, and long-term follow-up are warranted to validate and strengthen these findings.

The retrospective design and reliance on two-dimensional cephalometric analysis are inherent limitations of this study. The assessment of atypical swallowing was conducted clinically, without the use of electromyographic measurements. Additionally, although control subjects were matched for age and sex, they were drawn from a different population, potentially introducing variability due to genetic and environmental differences. To overcome these limitations future prospective studies incorporating three-dimensional imaging and long-term follow-up are essential to validate and confirm these findings.

## 5. Conclusions

Early intervention with the SOCIA III appliance showed potential improvements in sagittal skeletal relationships in patients with pseudo-Class III malocclusion and atypical swallowing. The device was associated with correction of anterior crossbite, improvements in overjet and overbite, and a possible positive influence on mandibular growth, while maintaining vertical facial proportions. By integrating functional re-education of tongue posture, SOCIA may offer a non-invasive interceptive approach addressing both skeletal and myofunctional aspects of malocclusion. These preliminary findings suggest that SOCIA III could represent a useful option for growing patients at risk of Class III functional malocclusion and for individuals with suspected orofacial myofunctional disorders. However, further randomized controlled trials with extended follow-up are needed to confirm these observations and establish their generalizability.

## Figures and Tables

**Table 1:** Cephalometric measurements.

Sagittal Growth	
SNA	The angle formed between the Sella–Nasion (SN) line and the Nasion–Point A line
ANB	The angle formed between the Nasion–Point A (NA) and Nasion–Point B (NB) lines. It is calculated by subtracting the SNB angle from the SNA angle
Wits	The perpendicular projection of Point A and Point B onto the functional occlusal plane
CB	The cranial base length, representing the anterior portion of the cranial base, is measured from Nasion (N) to Sella (S)
ACB	Anterior cranial base, the segment extending from the Foramen Caecum (Fc) to the Nasion (N)
Cephalometric Dental measurement	
Ui_PP	Maxillary incisor to PP plane: the angle formed between the long axis of the maxillary incisor and the palatal plane
Li-MP	Mandibular incisor to mandibular plane: the angle formed between the long axis of the mandibular incisor and the mandibular plane
Overbite	The vertical overlap of the maxillary central incisors over the mandibular central incisors, measured from incisal edge to incisal edge along the vertical axis
Overjet	The horizontal distance between the incisal edge of the maxillary central incisors and that of the mandibular central incisors, measured along the anteroposterior (sagittal) plane
Postural parameters	
CC Maxilla	Angle between odontoid process tangent through cvip and cv2tg (OPT) and PP
SNBa	The angle formed by the intersection of the Sella–Nasion (SN) line and the Nasion–Basion (N–Ba) line

**Table 2:** Descriptive statistics of the sample.

Descriptive Statistics										
	Group S (*n* = 52)					Group C (*n* = 52)				
	Mean	SD	Median	Min	Max	Mean	SD	Median	Min	Max
SNA_T0	81.56	1.29	81.10	80.23	82.85	81.52	1.46	80.80	79.30	83.01
SNA_T1	83.26	1.04	84.50	82.2	84.31	82.16	1.15	81.20	80.20	83.31
ANB_T0	0.61	0.32	0.75	0.29	0.94	0.45	1.1	0.87	−0.5	2.12
ANB_T1	2.06	0.3	1.62	1.76	2.37	−0.96	1.2	0.79	−2.90	1.40
Wits_T0	−0.75	0.87	−0.58	−1.62	0.12	−0.58	1.47	−0.23	−2.40	1.80
Wits_T1	1.24	0.36	1.05	0.87	1.61	−1.48	1.55	−1.80	−3.20	0.90
CB_T0	65.78	3.83	66.24	56.64	68.52	64.08	1.90	64.40	59.29	67.32
CB_T1	65.52	2.92	66.27	59.31	68.60	64.60	2.83	64.74	58.86	70.36
ACB_T0	9.36	2.75	9.38	6.01	16.07	10.62	2.29	10.61	7.38	15.23
ACB_T1	9.49	2.74	8.35	6.08	16.03	12.03	2.35	12.86	8.03	15.51
CCMaxilla_T0	92.76	8.34	94.26	79.55	106.53	90.68	7.05	91.74	79.53	102.84
CCMaxilla_T1	89.18	8.65	88.85	71.91	103.15	90.09	11.52	93.50	62.69	107.63
SNBa_T0	127.57	5.59	126.90	119.13	137.94	126.59	3.89	127.14	119.17	132.66
SNBa_T1	129.14	6.01	130.68	118.21	136.91	125.31	4.70	127.10	117.45	132.00
Ui_PP_T0	114.74	9.93	117.30	99.80	134	111.80	9.67	114.10	93.40	130
Ui_PP_T1	114.38	7.74	113.90	100.60	127.40	115.75	7.40	115	101.30	130.80
Li-MP_T0	96.11	7.84	97.70	83.90	110.30	87.25	6.36	89	75.10	98.50
Li-MP_T1	94.16	6.12	91.70	87.80	111.60	89.06	4.18	89.10	82.40	95.40
Overbite_T0	0.29	1.38	−0.10	−1.20	2.90	0.52	2.13	0.10	−2.60	5.60
Overbite_T1	1.82	0.79	2.00	0.40	3.30	1.27	2.37	0.30	−0.90	7.60
Overjet_T0	0.63	2.44	1.3	−3.30	3.80	0.93	2.23	1.30	−4	3.90
Overjet_T1	3.60	1.05	3.90	1.80	5	1.03	2.27	1.60	−3.90	4.10

**Table 3:** Comparison of cephalometric variables at To and T1 using paired *t*-test or Wilcoxon signed-rank test with Holm correction for multiple testing and Cohen’s d effect size (E.S.).

	Group S							Group C						
	T0		T1		P	HOLM CORR.	E.S	T0		T1		P	HOLM CORR.	E.S
	Mean	SD	Mean	SD				Mean	SD	Mean	SD			
SNA	81.56	1.29	83.26	1.04	0.006	**0.048**	1.45	81.52	1.46	82.16	1.15	0.11	N.S. (0.44)	0.49
ANB	0.61	0.3	2.06	0.3	0.001	**0.011**	4.83	0.45	1.1	−0.96	1.2	0.01	N.S. (0.11)	−1.22
WITS	−0.75	0.87	1.24	0.36	0.001	**0.011**	2.99	−0.58	1.47	−1.48	1.55	0.01	N.S. (0.11)	−0.6
CB	64.78	3.82	65.51	2.92	0.03	N.S (0.075)	0.21	64.08	1.89	64.40	2.82	0.16	N.S. (0.48)	0.13
ACB	9.35	2.75	11.45	2.74	0.05	N.S (0.125)	0.77	10.62	2.29	11.88	2.34	0.25	N.S. (0.50)	0.54
CCMAX	92.75	8.34	89.18	8.64	0.02	N.S (0.06)	−0.42	90.68	7.05	90.09	11.52	0.70	N.S. (0.70)	−0.06
SN-Ba	127.57	5.59	129.14	6.01	0.01	**0.055**	0.27	126.59	3.88	125.31	4.7	0.08	N.S. (0.48)	−0.3
UI-PP	114.73	9.93	114.38	7.74	0.81	N.S (0.81)	−0.04	111.89	9.67	115.74	7.4	0.009	N.S. (0.099)	0.45
LI-MP	96.11	7.84	94.16	6.11	0.13	N.S (0.26)	−0.28	87.25	6.36	89.06	4.18	0.07	N.S. (0.385)	0.34
OVB	0.29	1.38	1.82	0.79	0.001	**0.011**	1.36	0.52	2.13	1.26	2.36	0.01	N.S. (0.11)	0.33
OVJ	0.62	2.44	3.6	1.05	0.001	**0.011**	1.59	0.93	2.23	1.03	2.36	0.94	N.S. (0.94)	0.04

N.S. = not significant.

**Table 4:** Comparison of mean and SD values of cephalometric measurement between the group by *t*-test or Mann–Whitney test.

Cephalometric Measurement	Group S n = 52 Mean ± SD	Group C n = 52 Mean ± SD	SIG (2-Tailed)	Holm Correction	Effect Size (Cohen’s D)
SNA	1.69 ± 1.07	0.64 ± 1.23	0.12	N.S. (0.72)	0.91
ANB	1.46 ± 0.26	−1.2 ± 1.01	0.001 **	0.011	3.55
WITS	2 ± 0.74	−0.9 ± 1.15	0.001 **	0.011	2.95
CB	0.73 ± 1.91	0.49 ± 2	0.639	N.S. (1.00)	0.12
ACB	2.10 ± 1.24	1.26 ± 1.19	0.01 *	N.S. (0.08)	0.69
CCMAX	−3.57 ± 8	−0.16 ± 8.4	0.13	N.S. (0.78)	−0.42
SN-Ba	1.57 ± 3.27	−1.14 ± 3.9	0.002 **	0.02	0.75
UI-PP	−0.35 ± 7.5	3.4 ± 7.8	0.86	N.S. (1.00)	−0.49
LI-MP	−1.95 ± 8.28	1.39 ± 5.67	0.01 *	N.S. (0.07)	−0.47
OVERBITE	1.53 ± 1.79	0.88 ± 1.7	0.80	N.S. (1.00)	0.37
OVERJET	2.96 ± 2.27	0.17 ± 2.14	0.20	N.S. (1.00)	1.26

N.S. = not significant, ** *p* < 0.001, * *p* < 0.05

## Data Availability

The data presented in this study are available on request from the corresponding author due to privacy reasons.
